# Research Progress on the Gc Proteins of Akabane Virus

**DOI:** 10.3390/vetsci12080701

**Published:** 2025-07-27

**Authors:** Xiaolin Lan, Fang Liang, Gan Li, Weili Kong, Ruining Wang, Lin Wang, Mengmeng Zhao, Keshan Zhang

**Affiliations:** 1 Provincial Key Laboratory of Animal, Molecular Design and Precise Breeding, School of Animal Science and Technology, Foshan University, Foshan 528225, China; lan1041063917@outlook.com (X.L.); 15726370117@163.com (F.L.); ligan1227@163.com (G.L.); 2 Gladstone Institutes of Virology and Immunology, University of California, San Francisco, CA 94158, USA; 20220420224@stu.fosu.edu.cn; 3 College of Veterinary. Medicine, Henan University of Animal Husbandry and Economy; Zhengzhou 450046, China; 80882@hnuahe.edu.cn; 4 Institute of Cancer Sciences, University of Glasgow, Glasgow G12 80QQ, UK; 20220420206@stu.fosu.edu.cn

**Keywords:** Orthobunyavirus, the Simbu serogroup viruses, Akabane disease virus, Gc protein

## Abstract

The Akabane virus (AKAV) is a significant member of the Orthobunyavirus genus, with its envelope glycoprotein Gc, focusing on its molecular structural features, immunoregulatory mechanisms, and application value in pathogen diagnosis and vaccine design. As a key structural protein of AKAV, Gc mediates virus adsorption and neutralizing antibody recognition through the N-terminal highly variable region (HVR), while the C-terminal conserved region (CR) dominates the membrane fusion process, and its glycosylation modification has a significant regulatory effect on protein function. In clinical diagnostics, serological assays based on Gc proteins (e.g., ELISA, immunochromatographic test strips) have been standardized; in vaccine development, the neutralizing epitope of Gc proteins has become a core target for subunit vaccine design. Follow-up studies were deeply needed to analyze the structure-function interaction mechanism of Gc proteins to provide theoretical support for the construction of a new type of AKAV prevention and control system.

## Simple Summary

The Akabane virus (AKAV) is a significant arthropod-borne pathogen belonging to the *Orthobunyavirus* genus (family *Peribunyaviridae*), which poses a substantial threat to livestock health worldwide. Transmitted primarily by *Culicoide* midges, AKAV infects ruminants such as cattle and sheep, leading to severe reproductive disorders including abortion, stillbirth, and congenital deformities such as arthrogryposis-hydranencephaly syndrome (AHS). The viral envelope glycoprotein Gc is a key determinant of AKAV pathogenesis, serving critical functions in host cell attachment, membrane fusion, and immune evasion. This review systematically examines recent research progress on the structural and functional characteristics of the AKAV Gc protein. The unique domain architecture is examined in this review, with particular emphasis on the highly variable N-terminal region known to facilitate immune evasion and the conserved C-terminal region demonstrated to be essential for membrane fusion. The review also explores the role of Gc glycosylation in viral entry and immune modulation, as well as its interactions with host receptors such as heparan sulfate proteoglycans. Furthermore, we analyze how genetic variations in the Gc protein influence viral virulence, host adaptation, and cross-species transmission. In addition to basic virological aspects, this review highlights the translational applications of Gc protein research. Current serological diagnostic methods, including serum neutralization tests (SNT), enzyme-linked immunosorbent assays (ELISA), and colloidal gold immunochromatography, are summarized, with Gc-specific antibodies being leveraged for AKAV detection. The potential of Gc-based subunit vaccines is also discussed, with evidence showing that neutralizing epitopes in the Gc protein can elicit protective immune responses in animal models. By integrating molecular virology with applied research, this review provides comprehensive insights into the multifaceted roles of the AKAV Gc protein and underscores its importance as a target for diagnostics, therapeutics, and vaccine development. These advancements are critical for controlling AKAV outbreaks and mitigating its economic impact on the livestock industry.

## 1. Introduction

Members of the genus Orthobunyavirus are transmitted primarily by arthropods, and the viral particles are all spherical, 80–220 nm in diameter, have a vesicle membrane with spines, and a single-stranded RNA genome consisting of three segments, which replicate in the cytoplasm of the cells and assemble and bud from the membranes of the Golgi complex. These characteristics collectively form the cytobiological foundation of the Bunyaviridae [1]. Based on serological relationships, the Orthobunyavirus genus is divided into 18 serogroups, with the Simbu serogroup being one of the largest [2] and encompassing over 30 viral species. The serogroup contains more than 30 viruses. Based on the complete coding sequence of the M fragment, the more than 30 Simbu serogroup viruses were phylogenetically divided into two subgroups containing a total of seven virus complexes. Subcluster A contains two viruses, Oropouche and Manzanilla, while subcluster B contains five viruses, Akabane, Sathuperi, Shamonda, Simbu, and Shuni [3]. AKAV [4,5,6] belongs to the order Bunyavirales, family Peribunyaviridae, genus Orthobunyavirus, and the Simbu serogroup. This virus is not only capable of infecting ruminants but also has teratogenic effects and shares similar pathological symptoms with Schmallenberg virus (SBV) [7]. Furthermore, there is serological cross-reactivity between the two viruses [8]. Shin Murakami et al. [9] showed that heparan sulfate proteoglycans serve as the primary cellular attachment factors for AKAV and SBV, suggesting that these viruses may share similar mechanisms to facilitate their replication in susceptible cells. In comparison to other Bunyaviruses, such as Hantavirus, the high variability at the N-terminus of AKAV Gc protein and its cross-species adaptability make it an ideal model for studying viral evolution.

AKAV is primarily transmitted through bites from midges and can lead to abortion, premature birth, stillbirth, congenital malformations in newborns, and Arthrogryposis Hydranencephaly syndrome (AH) [10,11] in cattle and sheep. AKAV is widely distributed across Africa, the Middle East, East Asia, Southeast Asia, and Australia [12,13]. From 1972 to 1975, Japan reported over 31,000 cases of Akabane disease [14]. Australia confirmed more than 8000 cases of AH syndrome caused by AKAV in 1974 [15], and AKAV was first isolated from mosquitoes in Shanghai, China, in 1998 [16]. Subsequently, reports of AKAV distribution have emerged from Yunnan, Guizhou, Sichuan, and Guangdong provinces in China [17,18,19].

The Gc protein (also referred to as the G1 protein [20]) of AKAV serves as an envelope glycoprotein, and is not only a critical target for the immune system of vertebrate hosts but also capable of eliciting a strong, specific immune response. Additionally, it plays a decisive role in various important biological characteristics of AKAV, including pathogenicity, neutralization activity, hemagglutination ability, and cell fusion [21,22]. Therefore, this article aims to systematically summarize the structural characteristics, genetic variation patterns, immune regulatory mechanisms, and applications in diagnosis and vaccine development of AKAV Gc protein, to provide theoretical support for a comprehensive understanding of its pathogenic mechanisms and strategies for prevention and control.

## 2. Analysis of the Structural Characteristics and Functional Domains of the Gc Protein

### 2.1. Akabane Virus Structural Domain Composition

**Figure 1:**
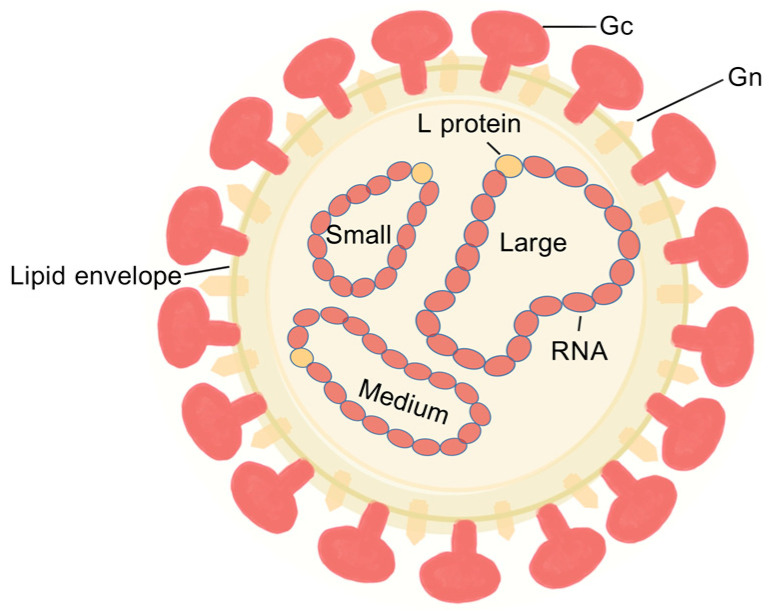
Schematic diagrams of AKAV.

Orthobunyaviruses possess three genomic segments (L, M, and S) [23,24], with the M segment encoding a polyprotein that cleaves into Gn, Gc, and NSm [21,25], showing high variability [26]. The glycoproteins Gn and Gc form viral surface spikes essential for attachment, membrane fusion, and immune response induction [27,28,29]. X-ray crystallography has elucidated the Gc protein’s 3D structure [30], advancing understanding of its receptor interactions and role in viral recognition and antibody neutralization [31,32] (Figure 1).

McPhee et al. [33] found that the structural proteins of all viruses within the Simbu serogroup of orthobunyaviruses exhibit typical *Bunyavirus* characteristics, particularly with the molecular weight ranges of the L, Gc, Gn, and N proteins being similar to those of Bunyamwera virus, where the molecular weight of the Gc protein ranges from 103 kDa to 125 kDa. The Gc protein is composed of structural domains from both the N-terminal and C-terminal ends, playing a crucial role in viral infection and pathogenicity [34]. The N-terminal of the Gc protein is located at the apex of the trimer and is exposed on the viral surface [35]; it consists of an α-helical bundle head and a rod-like “stalk” region. The head of the Gc protein contains critical glycan chains, and the low trimer affinity of the head facilitates the dissociation of the glycoprotein shell, allowing for cellular invasion. The semi-exposed three-dimensional structure of the Gc N-terminal serves as a precise target for humoral immunity [35], while the stalk region can be further divided into two subdomains. The variable region at the N-terminus of the Gc protein has become a primary target for host immune recognition and neutralizing antibodies due to its high immunogenicity and frequent mutations [35,36]. Yanase [21] and Hellert et al. [30] confirmed that the conserved Gc core domains, along with the three Gn ectodomains, are situated at the base, facilitating the connection of three adjacent spikes in close proximity to the viral membrane.

### 2.2. Gc Protein Glycosylation Modification

**Figure 2:**
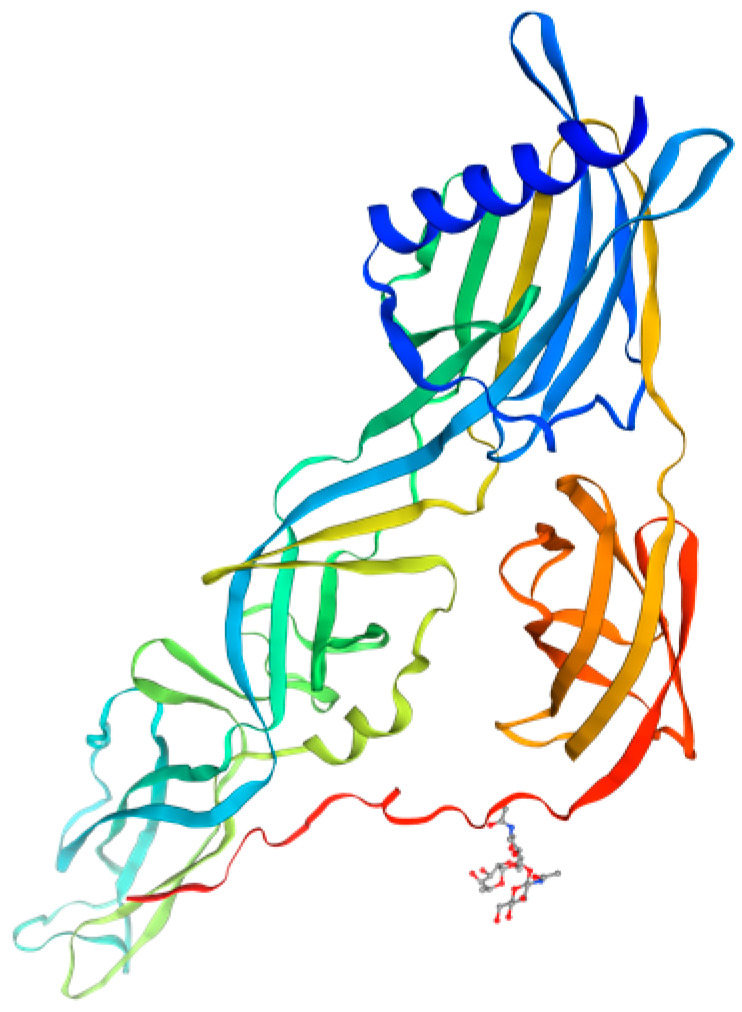
Pattern diagram of the tertiary structure of the Gc gene. The three-dimensional structure of the AKAV Gc gene was predicted using SWISS-MODEL online software. The amino acid sequence of the AKAV representative strain OBE-1 Gc was obtained from the NCBI database and imported into SWISS-MODEL for three-dimensional structure prediction.

The Gc glycoprotein of the Simbu serogroup viruses is classified as an I-type integral transmembrane (TM) protein and undergoes N-linked glycosylation modifications. It serves as the primary antigen for inducing neutralizing antibodies and has a wide range of functions [37], including recognizing host receptors, promoting membrane fusion for cellular invasion, regulating virulence, guiding viral morphogenesis, and possessing immune evasion capabilities, which facilitate replication and transmission within the host [38,39]. The glycosylation sites of the Gc protein were mapped onto its predicted tertiary structure (Figure 2). The SWISS-MODEL analysis demonstrated that N-linked glycans localize to the exposed regions of the α-helical head, consistent with their role in immune recognition [30]. The N-glycosylation sites of the Gc protein are conserved, and studies have shown that its glycan structure is complex. The degree of resistance of Gc proteins to digestion by endoglycosidase H highlights the importance of N-glycans for the proper folding and function of Gc glycoproteins [40,41,42]. Notably, while the glycosylation of the Gc protein is not essential for viral replication, it enhances viral growth; in contrast, Gn glycosylation is more critical for the correct folding of the glycoprotein [43].

### 2.3. Gc Protein Fusion Mechanism and Receptor Binding

Mutational experiments on the Gc cDNA conserved region have confirmed that this area functions as a fusion peptide. The fusion loop is a critical component of viral fusion proteins, particularly in I-type fusion proteins, where it is located at the N-terminus and is responsible for the initial insertion into the target membrane [44]. Experimental evidence indicates that the residues flanking the fusion peptide significantly influence the fusion conformation, with the deletion of these residues leading to failure in cell fusion [45]. Type II fusion glycoproteins of *Bunyaviruses* contain a fusion loop II composed of the cd, ij, and bc loops. In the Simbu serogroup viruses, the alanine at the end of the ij loop is adjacent to conserved amino acids that are essential for the functionality of the glycoprotein. Moreover, type II fusion glycoproteins of Bunyaviruses also contain a fusion loop II composed of the cd, ij, and bc loops [45,46,47,48]. In the Simbu serogroup, the alanine at the end of the ij loop is adjacent to conserved amino acids, which are essential for the functionality of the glycoprotein.

It is noteworthy that Shi et al. [49] have demonstrated that the cytoplasmic tail of the Gc protein also plays a significant biological role, with variations in this region affecting viral assembly and morphology. This further underscores the complexity and importance of the Gc protein and its components in the viral life cycle. During the viral life cycle, the membrane fusion process is mediated by the envelope glycoprotein Gc, which collaborates with Gn to form heterodimers [28,50]. The heterodimerization of Gn and Gc is also necessary for their transport to the Golgi apparatus [20,42]. The Golgi complex serves as the site for the assembly and maturation of Bunyaviruses through a budding process [29,51], serving as the core region for the generation and release of viral particles [52,53]. This complex assembly process enables viral particles to be effectively recognized by virus-specific neutralizing antibodies, which further influences the virus’s attachment to and invasion of host cells. This phenomenon has also been validated in other members of the *Bunyaviridae* family [54,55,56,57].

## 3. Immune Regulation and Pathogenic Mechanisms of the Gc Protein

### 3.1. Gc Protein Regulates Host Immune Response

The arrangement of these heterodimers allows every three Gn-Gc pairs to form a transmembrane trimer, creating a structure resembling the spiny trimer of coronaviruses. Gn-Gc heterodimers, especially Gc, are crucial in the viral life cycle by facilitating virus entry into cells and membrane fusion [30,58,59]. In addition, the Gc protein of AKAV is not only a key structural component of viral particles but also acts synergistically as a major target for neutralizing antibody responses through its variable and core conserved regions, highlighting its critical importance in the immune response. This feature is similarly expressed in other viruses of the Simbu serogroup, suggesting an important mechanism for Gc multifunctionality and host adaptation [30,35]. This observation is consistent with the essential role of Gn-Gc heterodimers, particularly Gc, in immune recognition and neutralization of viruses like RVFV [60,61]. However, significant differences exist in the ability to mount an immune response to the Gc protein among different hosts, attributable to variations in their immune systems, genetic backgrounds, and Gc protein expression characteristics. Ludwig et al. [62] further confirm that Gc plays a crucial role in the viral life cycle, particularly in neutralizing the virus and mediating its attachment to mammalian cells. This process is especially critical for the virus’s entry into host cells. The AKAV Gc protein has been identified to contain at least five antigenic regions, as demonstrated through competitive binding assays using neutralizing monoclonal antibodies [63]. This finding further enhances the understanding of the antigenicity of the Gc protein. The response characteristics of Gc-specific monoclonal antibodies, as revealed by Ogawa et al. [64], demonstrated that certain mutants, such as 1G4 and 1G7, exhibit immune responses akin to those of the wild-type virus. In contrast, mutants like 1A8 demonstrate differences in their binding to anti-Gc monoclonal antibodies, highlighting their unique immune recognition profiles. Roman-Sosa et al. [35] identified that the neutralizing epitopes of AKAV are located between positions 1 and 97. Hellert et al. [30] demonstrated through experiments on cattle and horses that the absence of the head-stalk structure of the Gc protein significantly reduces the reactivity of ELISA against the entire extracellular domain of Gc, nearly completely abolishing the serum’s neutralizing activity. This evidence indicates that the local antigenicity and immunogenicity within the glycoprotein spikes increase with the distance from the viral membrane. Studies have shown that antibodies targeting the viral envelope glycoproteins, particularly the Gc protein, possess the ability to neutralize the virus. In contrast, while anti-N antibodies are present in large quantities, they exhibit no neutralizing activity. The Gc protein is critical to the virus neutralization mechanism [65].

### 3.2. Effect of Gc Protein on Virus Replication

Experiments on the prototype Bunyavirus, BUNV, have confirmed that the first half of the Gc protein is crucial for viral replication under cell culture conditions [29]. Furthermore, additional studies involving the construction of deletion mutants revealed that viruses lacking this portion of the protein exhibit significantly impaired growth characteristics [66]. The Simbu serogroup viruses employ a transcription-translation coupling mechanism, in which the transcription of viral mRNA is highly dependent on continuous protein synthesis. Disruption of protein translation interferes with mRNA transcription, and inhibitors such as cycloheximide block viral replication, confirming that continuous protein synthesis is necessary for replication. Newly synthesized viral mRNA associates with ribosomes to prevent premature termination of transcription [67]. Barr et al. [68] revealed significant differences in replication efficiency among the three distinct segments of the BUNV genome, specifically showing that the M segment has the highest efficiency, followed by the L segment, while the S segment exhibits the lowest efficiency. This variation in efficiency may be related to the degree of complementarity in the terminal regions of each segment. Similar characteristics may also exist in AKAV. AKAV transcription and translation rely on the L protein and the N protein. Although detailed reports on its gene replication and expression are limited, studies on the prototype Bunyavirus reveal that after the viral genome enters the cytoplasm, it migrates to the rough endoplasmic reticulum (RER). The L protein facilitates the transcription of vRNA into cRNA and utilizes a “cap-snatching” mechanism to employ host mRNA 5′ oligoadenylates as primers. Some of the cRNA then associates with newly synthesized L and N proteins to form cRNPs, which, under the action of the L protein, replicate to generate progeny vRNPs, thereby amplifying the replication of the Simbu serogroup viruses. The Gc protein may work in concert with the L and N proteins to influence the efficiency of viral gene replication and expression, thereby regulating the overall replication level of the virus [69]. Additionally, a portion of the cRNA does not associate with the L and N proteins but instead binds to ribosomes on the RER to undergo the translation process [70].

### 3.3. Effect of Gc Protein on Virulence

**Figure 3:**
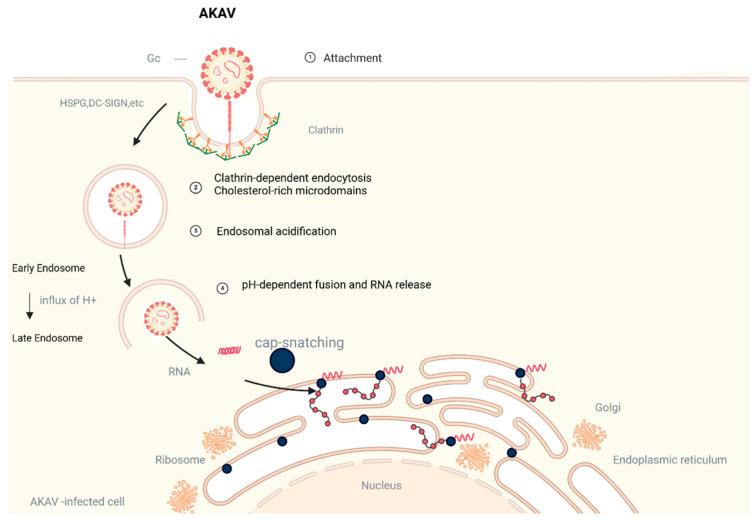
AKAV Infection model. Virus Attachment: AKAV binds to host cell surface receptors (e.g., HSPG, DC-SIGN, and Axis) via membrane interactions. Endocytosis: Viral entry occurs through clathrin-dependent endocytosis or cholesterol-rich microdomains (lipid rafts). Endosomal Acidification: Influx of H+ in early endosomes triggers acidification, promoting pH-dependent fusion of the viral envelope with the endosomal membrane and subsequent RNA release. RNA Release and Cap-Snatching: Within late endosomes, the virus employs a “cap-snatching” mechanism to hijack host mRNA caps, initiating viral RNA transcription. Viral Replication: Viral RNA is translated into proteins by ribosomes, with some proteins processed in the endoplasmic reticulum. Genome Trafficking: Viral genetic material may enter the nucleus for replication, ultimately forming an AKAV-infected cell.

The Gc proteins of AKAV promote viral attachment and invasion by binding to host cell surface receptors. Gc-mediated viral entry mechanisms are similarly characterized in Orthobunyavirus (OBV) and other viruses of the Simbu serogroup. In studies of AKAV, mutation experiments with Gn and Gc proteins further validated that high-affinity binding of specific receptor-binding regions of Gc to HS is critical for viral invasion [71,72]. In addition, the Gc protein of AKAV triggers receptor-mediated endocytosis through its conserved region when interacting with HS and other host molecules, thereby facilitating viral entry into cells. Thus, the Gc proteins of OBV and AKAV share a high degree of functional congruence in the mechanism of viral invasion. Studies have shown that lectin-mediated endocytosis plays an important role in OBV virus invasion. In addition, AKAV studies further revealed its mechanism of cellular entry via the dynamin- and lectin-dependent endocytosis pathways, and experimentally verified the inhibitory effect of lectin inhibitors on viral invasion [73]. Notably, the Gc protein of AKAV not only initiates endocytosis through binding to host surface molecules, but also requires the support of cholesterol and other lipid microregions to facilitate efficient uptake of viral particles [74]. This mechanism suggests a remarkable functional similarity between the Gc proteins of OBV and AKAV in utilizing the host cell endocytosis pathway, further highlighting the central role of the Gc proteins in the viral invasion process. Gc preconditioning under endosomal acidification conditions plays an important role in the invasion of both OBV and AKAV viruses. It has been shown that the fusion of the viral envelope with the cell membrane is the final step in the entry of the enveloped virus into the host cell, allowing the release of the viral genome into the cytoplasm [74]. In studies of AKAV, conformational changes in Gc proteins were dependent on the triggering of endosomal acidification, a process that is regulated by conserved histidine residues in Gc and determines the optimal pH for viral fusion [75]. In addition, both AKAV and OBV infections are highly sensitive to drugs that alter endosomal pH (e.g., chloroquine and NH4Cl), further suggesting a key role for endosomal acidification in viral invasion [74] (Figure 3).

Sequence variations in these proteins may affect the efficiency of viral infection and the range of hosts. Mutations in the immunogenic regions of the Gc protein may trigger immune evasion, facilitating the persistence and spread of the virus within the host. Studies have shown that the deletion or mutation of the Gc proteins of AKAV reduces virulence while still eliciting an immune response from the host [31,35]. Moreover, the sequence diversity in the highly variable N-terminal region of Gc (e.g., low sequence similarity between Schmallenberg virus, La Crosse virus, Bunyavirus (SBV, LACV, BUNV) enables the virus to evade host immunorecognition, presenting challenges for antibodies to neutralize a wide range of different strains. [30,59,76]

## 4. Serological Diagnostic Techniques for AKAV Gc Protein

### 4.1. The Serum Neutralization Test

The serum neutralization test (SNT) is recognized as the standard technique for the diagnosis of antibodies to bovine and ovine AKAV and is highly specific. The principle of the test is that when the virus binds to the corresponding antibody, it loses its ability to infect cells, and the potency of the antibody is determined by counting the number of cell lesions. The quality of the antigen and the standard positive serum is a key factor in determining whether the SNT can be successfully performed. Liu [77] used the AKAV OBE-1 strain to successfully prepare antigen and high-immunity serum, and based on this, they constructed the AKAV micro-serum neutralization test system, which achieved good results in practical application. However, the SNT is not suitable for testing large amounts of animal serum samples because of its cumbersome operation procedure, susceptibility to cell contamination, and time-consuming detection.

### 4.2. The Enzyme-Linked Immunosorbent Assay

The enzyme-linked immunosorbent assay (ELISA) has been widely used in the serological detection of Acanthamoeba virus (AKAV) due to its significant advantages, such as high sensitivity, ease of operation, and the ability to process a large number of samples. Yohsuke Ogawa et al. [78] identified two neutralizing epitope domains in the AKAV Gc protein (Gc1–97 and Gc189–397), which are capable of inducing the production of neutralizing antibodies in mice. This suggests that these epitope domains have the potential to serve as effective subunit vaccine candidates. Similarly, studies on RVFV have shown that Gn-Gc heterodimers, especially Gc, play a crucial role in virus entry and immune recognition.) In addition, the heterodimers formed by Gn-Gc constitute the viral capsid, whereas Gc-Gc contacts may form a Gn-independent inner shell responsible for viral membrane fusion [60,61]. These findings highlight the importance of Gc in the viral entry process and suggest that targeting Gc may be a promising strategy for diagnostics and vaccine development. Commercial diagnostic tools utilizing Gc protein epitopes have been developed to facilitate AKAV detection. For example, IDvet (France) has developed a commercialized ID screen AKAV antibody detection kit using a competitive ELISA method, which is currently recognized as a highly specific and sensitive AKAV antibody detection kit, but there are no commercialized serological diagnostic kits available in China [79,80,81,82]. Compared to serum virus neutralization tests (SNT), these kits reduce turnaround time and are suitable for large-scale surveillance. Wang et al. [83] developed three monoclonal antibodies against the AKAV Gc protein (4D1, 4E6, and 4F12) and identified a broadly neutralizing epitope located on the Gc protein (1134SVQSFDGKL1142). This epitope exhibits high conservation across different AKAV genotypes, offering new possibilities for the development of a broad-spectrum vaccine.

In the field of monoclonal antibody production, Wei et al. [84] successfully expressed a critical fragment of the AKAV Gc protein. This fragment is referred to as (Gcaa465~704) and was produced using an insect cell baculovirus expression system. They screened hybridoma cells generated from immunized mice to identify those capable of stably secreting a monoclonal antibody against this protein (mAb 4D1). Additionally, Wang et al. [85] achieved efficient soluble expression of AKAV Gc_405aa-480aa_ peptides using a prokaryotic expression system. They purified and characterized the antigenicity of this recombinant peptide, providing a foundation for the subsequent development of monoclonal antibodies and diagnostic reagents. The development of these monoclonal antibodies targeting the Gc protein opens new avenues for the treatment of viral infections, as these antibodies can precisely neutralize the virus, curb its spread, and mitigate pathological damage.

### 4.3. Colloidal Gold Immunochromatography

Colloidal gold immunochromatography is a new immunological diagnostic technique developed based on the principles of colloidal gold labeling, immunodetection, and chromatographic analysis [86]. The core of the technique is the specific binding of colloidal gold-labeled antibodies to target antigens, resulting in visual color changes in test strips to detect specific antigens. The advantages of this technique are outstanding, not constrained by the detection environment, no need for precision instruments, low cost, and economic and environmental protection, and the results are judged intuitively, which is suitable for on-site testing of large numbers of samples. Zhang [87] created the spot immunogold filtration method, which uses colloidal gold to label SPA for color development, with good specificity, easy operation, and without special equipment, and is suitable for import and export quarantine and grass-roots animal epidemic prevention. Yang [88] developed a rapid, stable, and easy-to-use test strip for the detection of Acanthamoeba disease with high sensitivity and specificity, but the detection rate was poor due to the difficulty of virus sampling. Kong [89] constructed AKAV antibody test strips, and the results were obtained within 10 min of serum sample collection, which fit the needs of rapid screening in the field. However, the sensitivity of colloidal gold technology is relatively insufficient, and there is a risk of false negatives [74]. In the future, it is necessary to improve the quality of colloidal gold and integrate new molecular markers and other cutting-edge technologies to break through the existing limitations.

### 4.4. Indirect Fluorescent Antibody Technology

Li [90] pioneered the construction of an indirect immunofluorescence technique for the specific detection of AKAV antigens. This technique uses rabbit anti-AKAV recombinant protein as a primary antibody, which is combined with FITC-labeled goat anti-rabbit IgG for carrying out the detection. This method has the advantages of high specificity, fast operation, and no interference from parent antibody, but the operation procedure is relatively complicated and depends on hardware detection equipment, which limits its popularization and application in the field of rapid detection to a certain extent.

## 5. Challenges and Prospects of Utilizing the Gc Protein in Vaccine Research

Currently, progress has been made in the development of vaccines targeting the envelope glycoprotein Gc of both AKAV, yet multiple challenges remain. In the context of AKAV vaccine development, the application of reverse genetics has significantly advanced the research of potential attenuated vaccine candidates. However, most studies are still limited to laboratory settings, and further investigation is needed regarding the commercialization process, as well as the validation of protective efficacy and safety. The AKAV Gc protein, containing neutralizing epitopes, is considered a potential target for subunit vaccine development. Yohsuke Ogawa et al. [78] successfully expressed the AKAV Gc protein using a baculovirus system and validated its immunogenicity and ability to induce neutralizing antibodies in mice. To further advance the practical application of the vaccine, it is essential to explore its immunoprotective effects in ruminants. Wang et al. [83] found that the neutralizing epitope in the Gc protein is highly conserved across different genotypes of AKAV, providing feasibility for the development of diagnostics and vaccines targeting multiple AKAV strains. The N-terminal domain of the SBV Gc protein has been confirmed to be antigenic, indicating its ability to stimulate the host’s immune response. Given that this domain can be recognized by neutralizing antibodies, it is regarded as an important target in vaccine development [35]. Vaccines incorporating this domain can effectively induce an immune response against SBV in the host [36]. Boshra et al. [91] developed DNA and recombinant vaccines targeting the N-terminus of the Gc protein, demonstrating good immunogenicity and protective efficacy. Wernike et al. [92] conducted vaccination-challenge experiments in cattle using recombinant EHV-1 or MVA vector viruses to deliver the N-terminal domain. The results showed that the EHV-1-based vaccine was partially effective, whereas the MVA vector vaccine induced SBV-specific antibody responses, providing complete protection. These vector vaccines are useful for distinguishing between vaccinated animals and those infected in the field. Wernike et al. [93] constructed a polyvalent vaccine containing both SBV and related AKAV Gc domains, demonstrating that all vaccinated cattle and mice were completely protected from SBV infection. This highlights the potential of the Gc protein N-terminus in vaccine development and showcases the superior efficacy of polyvalent vaccines in providing protection. The Gc protein on the surface of the virus serves as a key antigen, and in-depth studies of its immunogenicity are crucial for vaccine development. The Gc protein effectively induces the host to produce neutralizing antibodies, which provide effective protection by blocking the virus’s ability to bind to host cells. This underscores the significance of Gc protein-based vaccines in the field of virology research.

## 6. Summary and Future Prospect

The Gc proteins of AKAV, as key membrane proteins of the orthobunyavirus genus, are significant for understanding the mechanisms of viral pathogenicity, immune responses, and vaccine development due to their structure, function, and genetic variability. The Gc protein consists of an N-terminus and a C-terminus, with the N-terminus located at the apex of the viral trimer and exposed on the virus surface. It is composed of an α-helical bundle head and a rod-like “stalk” region. The head contains important glycan chains, and the unique structure of the Gc N-terminus not only facilitates the interaction between the virus and host cells but also plays a critical role during the viral entry into cells. The Gc N-terminus also exhibits high variability and serves as a primary target for neutralizing antibodies, while the C-terminus is relatively conserved and primarily functions in membrane fusion. The Gc protein plays a crucial role in viral attachment, membrane fusion, and Golgi localization through its complex glycosylation modifications and transmembrane structure. When transmitted from arthropods to mammals, the Simbu serogroup viruses’ Gc protein necessitates rapid further evolution through genetic recombination and mutation to adapt to the mammalian host environment. This adaptation includes the potential necessity for the Gc protein to evolve new structural domains or modify existing ones to bind to mammalian cell surface receptors (such as Lrp1 or HS). The immune system of mammals being more complex, the Gc protein may evolve new glycosylation sites or other modifications to evade immune recognition in mammals. Optimization of the membrane fusion efficiency of the Gc protein in mammalian cells may be required to ensure the virus can effectively enter cells and initiate replication. The adaptive evolution of the Gc protein directly impacts the increased transmission efficiency and enhanced pathogenicity of the Simbu serogroup viruses. In summary, the adaptive evolution of the Gc protein in arthropods and mammals is a key factor in the interspecies transmission and host adaptation of the Simbu serogroup viruses. In-depth research into its evolutionary mechanisms will provide crucial insights for controlling the spread of the Simbu serogroup viruses and developing effective vaccines. [94] Its genetic variability not only affects the virus’s antigenicity and pathogenicity but also determines its adaptability and transmissibility. The immunogenicity of the Gc protein is significant, as it can elicit a strong immune response in the host while also potentially leading to immune evasion. Additionally, the Gc protein can directly influence viral replication and proliferation. It interacts with viral RNA and nucleoproteins through its zinc finger domain, collectively facilitating the formation of viral particles.

Current research focuses primarily on the structure and function of the Gc protein, its genetic variability, and mechanisms of immune evasion, as well as its role in viral replication and pathogenicity. Particularly due to the high variability of its N-terminus, the Gc protein can evade host immune recognition, which is a key factor in the virus’s ability to persistently infect and spread. However, this variability may also reduce the virus’s replication efficiency and have complex effects on its pathogenicity and transmission range. The Gc protein is involved not only in the processes of viral attachment and entry but may also influence viral replication efficiency. Nevertheless, the specific mechanisms by which the Gc protein regulates viral replication still require further investigation. Although the structural features of Gc proteins are well characterized, the specific relationship between their structure and function—particularly how N-terminal variability affects viral function and immune evasion mechanisms—still requires further investigation. Future studies on the structure and function of the Simbu serogroup viruses. Gc proteins are expected to advance in a more in-depth and comprehensive manner. For example, high-resolution structural biology techniques, such as X-ray crystallography and cryo-electron microscopy, can be employed to further elucidate the fine structure and functional characteristics of the Gc protein, particularly the impact of N-terminal variability on viral function and immune evasion mechanisms. By utilizing gene editing and molecular biology techniques, the regulation of the viral replication process by the Gc protein, as well as the impact of this regulation on the virus’s pathogenicity and transmission range, warrants further investigation. Based on a deep understanding of the structure and function of the Gc protein, specific antiviral drugs and vaccines targeting the Gc protein can be designed and developed. This provides new strategies and methods for the prevention and control of the Akabane virus infections.

## Data Availability

No new data were created or analyzed in this study. Data sharing is not applicable to this article.
